# Fetal gender and gestational age differentially affect PCSK9 levels in intrauterine growth restriction

**DOI:** 10.1186/s12944-016-0365-6

**Published:** 2016-11-14

**Authors:** Ulrich Pecks, Werner Rath, Nicolai Maass, Bartlomiej Berger, Imke Lueg, André Farrokh, Sabrina Farrokh, Christel Eckmann-Scholz

**Affiliations:** 1 Department of Obstetrics and Gynecology, University Hospital of the RWTH, Aachen, Germany; 2 Department of Obstetrics and Gynecology, University Hospital Schleswig-Holstein, Kiel, Germany

**Keywords:** Intrauterine growth restriction (IUGR), PCSK9, cholesterol metabolism, LDL receptor, LDL-C

## Abstract

**Background:**

Maternal and fetal Low Density Lipoprotein-Cholesterol (LDL-C) concentrations are compromised in intrauterine growth restriction (IUGR). Generally, LDL-C catabolism is under control of PCSK9 by binding to the LDL-receptor leading to its degradation. Hence, we hypothesized a role for PCSK9 in the modulation of lipid metabolism and placental transport in IUGR.

**Methods:**

172 women, 70 IUGR and 102 controls were included in the study. Maternal and fetal serum PCSK9 levels and lipid profiles including LDL-C were measured. Placental LDL-receptor and PCSK9 expressions were estimated by tissue microarray immunohistochemistry, and analyzed by two blinded observers using an immunoreactivity score. Non-parametric tests and multivariate regression analyses were used for statistical estimations.

**Results:**

PCSK9 levels in the maternal and fetal compartment independently predicted LDL-C levels (maternal compartment: adjusted *R* ^2^ = 0.2526; coefficient *b*_*i*_ = 0.0938, standard error s_*bi*_ =0.0217, r_partial_ = 0.4420, *t*-value = 4.323, *p* < 0.0001; fetal compartment: adjusted *R* ^2^ = 0.2929; *b*_*i*_ = 0.1156, s_*bi*_ =0.020, r_partial_ = 0.5494, t-value = 5.81, *p* < 0.0001). We did not find significant differences in maternal PCSK9 concentrations between IUGR and controls. However, we found lower fetal serum PCSK9 concentrations in IUGR than in controls (IUGR median 137.1 ng/mL (95% CI 94.8-160.0) vs. controls 176.8 (154.6-202.5), *p* = 0.0005). When subgrouping according to early onset, late onset IUGR, and fetal gender differences remained consistent only for male neonates born before 34 weeks of gestation. In the placenta we found no correlation between PCSK9 and LDL-receptor expression patterns. However, the LDL-receptor was significantly upregulated in IUGR when compared to controls (*p* = 0.0063).

**Conclusions:**

Our results suggest that PCSK9 play a role in impaired fetal growth by controlling fetal LDL-C metabolism, which seems to be dependent on gestational age and fetal gender. This underlines the need to identify subgroups of IUGR that may benefit from individualized and gender-specific pharmacotherapy in future studies.

## Background

Pregnancy is associated with immense changes in lipid metabolism. Maternal blood concentration levels of the predominant cholesterol transport vehicles, the Low-Density Lipoproteins (LDL), increase by about 70% from first to third trimester [1]. As a precursor of steroid hormones the placenta requires LDL-Cholesterol (LDL-C) for the synthesis of progesterone [2]. Moreover, cholesterol is an essential component for placental and fetal development, and it is estimated that 20 to 50% of fetal cholesterol originates from the mother by active transplacental cholesterol transport [3, 4]. The uptake of LDL by the placenta is well described and particularly involves the LDL-receptor (LDLR) [3].

Compromised fetal development occurs in 3 to 8% of all pregnancies, and is associated with increased perinatal morbidity and mortality because of fetal hypoxia or iatrogenic preterm delivery [5].

Recently, we reported corresponding to the results of others that intrauterine growth restriction (IUGR) is linked to a disturbed cholesterol metabolism associated with lower LDL-C concentrations in maternal and fetal blood [6–9]. The mechanisms regulating cholesterol homeostasis during pregnancy and under pathological conditions such as IUGR are not fully understood.

Proprotein Convertase Subtilisin/Kexin-Type 9 (PCSK9) is a serine protease secreted as a glycoprotein into the circulation. Since its discovery in 2003 PCSK9 has emerged as a significant modulator of LDL metabolism [10, 11]. Loss of function mutations of PCSK9 are associated with low plasma LDL-C levels [12, 13]. The mode of action of PCSK9 is its binding to the LDLR subsequently leading to internalization and degradation of the receptor [14]. As a consequence the reduced number of LDLR on the cell surface leads to lower LDL turnover rates and higher circulating LDL-C concentrations [15]. The inhibition of the mechanism lowers LDL-C levels, and this has led to the development of PCSK9 inhibitors like evolocumab which have been approved for the treatment of hypercholesterolemia in 2015 when statine therapy fails.

Recent studies confirmed the presence of PCSK9 in maternal and umbilical cord blood as well as in the placenta [16–18]. Indeed, concentration levels of PCSK9 were higher in pregnant women at term than in non-pregnant women suggesting a potential role in controlling cholesterol homeostasis during pregnancy [17].

Since LDL metabolism is disturbed in IUGR we hypothesized that PCSK9 is involved in the regulation of LDL distribution throughout different compartments (maternal, placental, and fetal) and that it contributes to the pathogenesis of IUGR by affecting transplacental cholesterol transport. Because therapeutic options already exist, the PCSK9/LDL metabolism seemed a promising pathway to be studied in pregnancy pathologies.

## Methods

It was the aim of the present study to compare PCSK9 levels in maternal and fetal blood of pregnancies complicated by IUGR and uncomplicated pregnancies, and to investigate the expression patterns of PCSK9 and its ligand, the LDLR, in the placenta. Hence, biomaterial for this case-control study was sampled prospectively taking into account the three different compartments of the materno-placento-fetal unit. Prior to the study, a power analysis was performed by calculating the minimal required sample size on the basis of the maternal LDL-C/High Density Lipoprotein-Cholesterol (HDL-C) ratio. Choosing a power (1–β-error) of 80% and a level of significance (α-error) below 0.05 for each group, IUGR and CTRL, at least 26 patients had to be included [7].

### Study cohort

Biomaterial was sampled at the Department of Obstetrics and Gynecology, University Hospital of the RWTH Aachen between July 2006 and March 2012 as described [19–21].

Clinical data were recorded at inclusion and following delivery. Gestational age was calculated by the last menstrual period and verified by first trimester scan documentation as described [7]. Maternal blood was taken after 24 weeks of gestation and prior to delivery. Fetal cord blood and placental tissue were sampled immediately after birth.

172 individual patients were selected. Of those 70 patients were classified as IUGR and 102 served as controls (CTRL) with adequate growth and birthweight between 10^th^ and 90^th^ percentile. In 54 patients of the IUGR group iatrogenic delivery was performed preterm before 37^th^ week of gestation because of non-reassuring fetal wellbeing. 51 patients of the CTRL group delivered preterm because of onset of spontaneous preterm delivery or premature rupture of the membranes. Important clinical characteristics include maternal age, BMI, blood pressure, fetal birth weight, and birth weight centiles (Table 1). Maternal lipid profile including LDL-C, HDL-C, total cholesterol (TC) and triglyceride (TG) values were available in 99 patients of the CTRL group and in 70 patients of the IUGR group. Fetal lipid profile was measured in 76 CTRL and 54 IUGR cases. Fetal and neonatal birth weight centiles were determined according to the population-based, newborn weight charts [22]. Additionally, customized centiles were calculated by the use of the online platform www.gestation.net (Gardosi J, Francis A. Customised weight centile calculator. GROWTH V6.7.6.11 (DE)).

**Table 1:** Maternal and neonatal clinical characteristics and lipid concentrations

	CTRL			IUGR			P
	n	Mean or %	95% CI	n	Mean or %	95% CI	
maternal age (y)	102	31.9	30.7–33.0	70	29.6	28.1–31.2	**0.0193**
maternal hight (m)	102	1.67	1.66–1.69	70	1.66	1.64–1.67	0.0878
maternal pre-pregnancy weight (kg)	102	66.4	63.3–69.5	70	67.6	63.5–71.6	0.6388
maternal pre-pregnancy BMI (kg/m^2^)	102	23.7	22.7–24.6	70	24.5	23.2–25.8	0.2686
maternal parity	102	0.95	0.75–1.16	70	0.64	0.37–0.91	0.0682
maternal smoking status (%)	102	20	12.0–27.0	70	36	24.0–47.0	**0.0181**
maternal pre-pregnancy smoking status (%)	102	39	30.0–49.0	70	47	35.0–59.0	0.3042
maternal systolic blood pressure (mmHg)	102	117	114.6–119.0	70	127	122.7–131.9	**0.0000**
maternal diastolic blood pressure (mmHg)	102	67	64.9–68.5	70	76	72.2–78.8	**0.0000**
hypertension in pregnancy (%)	102	0	0.0–0.0	70	27.0	16.0–38.0	**0.0000**
gestational age at inclusion	102	32.2	31.2–33.1	70	31.6	30.5–32.6	0.4053
delivery week of gestation (w)	102	35.3	34.4–36.2	70	32.7	31.7–33.7	**0.0002**
neonatal gender (% female)	102	49	39.0–58.9	70	46	34.0–58.0	0.6720
neonatal weight at birth (g)	102	2559	2376–2743	70	1347	1192–1502	**0.0000**
neonatal birth weight centile (population based)	102	43.7	39.8–47.6	70	4.1	3.5–4.8	**0.0000**
neonatal birth weight centile (customized)	102	43.1	38.2–48.0	70	0.7	0.2–1.1	**0.0000**
maternal LDL cholesterol (mg/dL)	99	152.5	144.6–160.5	70	125.5	114.6–136.4	**0.0001**
maternal HDL cholesterol (mg/dL)	99	75.6	72.1–79.1	70	71.9	67.88–75.92	0.1694
maternal total cholesterol (mg/dL)	99	268.6	258.6–278.7	70	235.6	222.6–248.6	0.0001
maternal triglycerides (mg/dL)	99	238.3	216.7–259.9	70	214.4	189.5–239.3	0.1525
fetal LDL cholesterol (mg/dL)	76	28.5	25.1–31.9	54	20.54	17.01–24.06	**0.0018**
fetal HDL cholesterol (mg/dL)	76	31.0	28.9–33.0	54	15.81	13.84–17.79	**0.0000**
fetal total cholesterol (mg/dL)	77	70.1	65.4–74.9	54	50.78	46.37–55.18	**0.0000**
fetal triglycerides (mg/dL)	77	22.4	19.6–25.1	54	45.19	37.65–52.72	**0.0000**

*P*-values are in bold if considered significant (<0.05)

IUGR was defined in accordance to the ACOG guidelines as described recently [5, 7]. In addition to having an estimated fetal weight below the 10^th^ centile, one of the following criteria had to be fulfilled: (i) deceleration of fetal growth velocity during the last 4 weeks, (ii) elevated resistance index in umbilical artery Doppler sonography above the 95th percentile or absent or reversed end-diastolic blood flow (ARED), (iii) fetal asymmetry (head to abdominal circumference ratio above the 95th percentile), or (iv) oligohydramnios (amniotic fluid index <6 cm). Patients were further subgrouped into early onset (before 34 weeks) and late onset (after 34 weeks) phenotype of IUGR [23, 24].

Exclusion criteria were the following: multiple gestation, fetal anomalies, abnormal fetal karyotype, patients with clinical or biochemical signs of infection, positive TORCH screening results, maternal diabetes mellitus/gestational diabetes or other severe maternal metabolic disorders, as well as patient’s withdrawal from the study.

### Blood sampling, serum generation and storage of aliquots

Blood samples were taken using Monovette syringes (Serum 4.9 mL Monovette, Sarstedt, Germany). After incubation at room temperature for 15–30 min, samples were centrifuged at 2000 *g* for 15 min. Serum was aliquoted and stored at −80 °C.

### Basic serum lipid profiling

TG, TC, LDL- and HDL-C levels were measured by colorimetric enzymatic methods using an automated photometric measuring unit (Roche/Hitachi Modular P800, Roche Diagnostics, Basel, Switzerland) as described [7].

### Enzyme Linked Immunosorbant Assay (ELISA)

PCSK9 concentrations were determined by enzyme linked immunosorbent assays (ELISA) (Biorbyt, Cambridge, UK) in duplicates according to the manufacturer’s protocol.

### Placental sampling, tissue microarrays and immunohistochemistry

Random pieces of the intermediate third of the placenta were chosen from vital cotyledons that were macroscopically free of infarct areas or other obvious morphologic pathologies immediately after delivery. Processing and paraffinisation was done according to a standardized protocol as described [21]. Of each individual patient three placental tissue samples were then rearranged in tissue microarray paraffin blocks achieving a total of 40 IUGR and 40 gestational age matched CTRL triplicates. Immunohistochemistry was performed as previously described [21, 25] using polyclonal goat anti-human PCSK9 (1:40), and monoclonal mouse anti-human LDLR (1:400) antibody (Novus, Littleton, CO). Sections were counterstained with hematoxylin. Control reactions excluding the use of primary antibodies did not reveal any staining. A positive control was done using human hepatic tissue. Two blinded observers (BB and IL) selected the central high power field (1:400) of each placental tissue spot using an All-in-one Microscope Modell BZ-9000E HS (Keyence, Osaka, Japan) equipped with a BZ-H2AE Image Analyzer. Evaluation of staining pattern and intensity were performed using immunoreactivity score (IRS) separately for trophoblasts and endothelial cells. Interobserver variability was below 10%.

### Statistics

nQuery Advisor 7.0 (Janet D. Elashoff (2007), CA, USA) was used for sample size calculation. Data analysis was carried out using the statistical packages Prism Version 6.0e Software (GraphPad Software Inc., CA, USA). Clinical data are presented as means ± 95% CI. Analytical variables are expressed as median ± 95% CI. Two-tailed Mann-Whitney-Test was conducted for comparison of metric variables. Fisher’s exact test was used for categorical data. Correlations were analyzed by Spearman’s correlation coefficient. Values of *p* < 0.05 were regarded as significant. Stepwise multivariate linear regression analyses were performed to assess the association between serum levels of PCSK9 and LDL-C, and gestational age at sampling and delivery, fetal gender, and study-group. Logistic regression analysis was performed to estimate the predictive value of PCSK9 and LDL-C on the diagnosis of IUGR using MedCalc® V9.4.2.0. The variables with a *p*-value < 0.1 on univariate analysis were entered into multivariate models.

## Results

### Clinical characteristics and lipid profiles of the study cohort

Maternal and neonatal characteristics are summarized in Table 1. Maternal age and smoking status during pregnancy differed slightly between the study groups while pre-pregnancy BMI, parity, gestational age at inclusion into the study, and neonatal gender were kept similar. None of the CTRL group and 19 women (27%) in the IUGR group were hypertensive during pregnancy. Gestational age at delivery was kept similar in patients in which placenta (CTRL mean 32.5 weeks (95% CI 31.2–33.8) vs. IUGR 32.0 (30.8–33.1)) and/or fetal cord blood (CTRL mean 34.2 weeks (95% CI 32.9–35.5) vs. IUGR 34.0 (32.7–35.3)) had been analyzed. For analyzes of maternal blood, only those patients were included in the CTRL group who delivered a healthy neonate after 37 weeks of gestation to avoid masking pathologic conditions (weeks of gestation at sampling: CTRL mean 31.5 weeks (95% CI 30.0–33.0) vs. IUGR 31.1 (29.7–32.6), weeks of gestation at birth: CTRL mean 38.8 weeks (95% CI 38.3–39.3) vs. IUGR 31.8 (30.4–33.3)). Mean maternal LDL-C concentration was 18% lower in IUGR as compared to CTRL. Mean TC concentration was 12% lower. In the fetal compartment LDL-C and TC concentrations were lower by 28% in IUGR as compared to CTRL (Table 1).

### PCSK9 in the maternal compartment

**Fig. 1:**
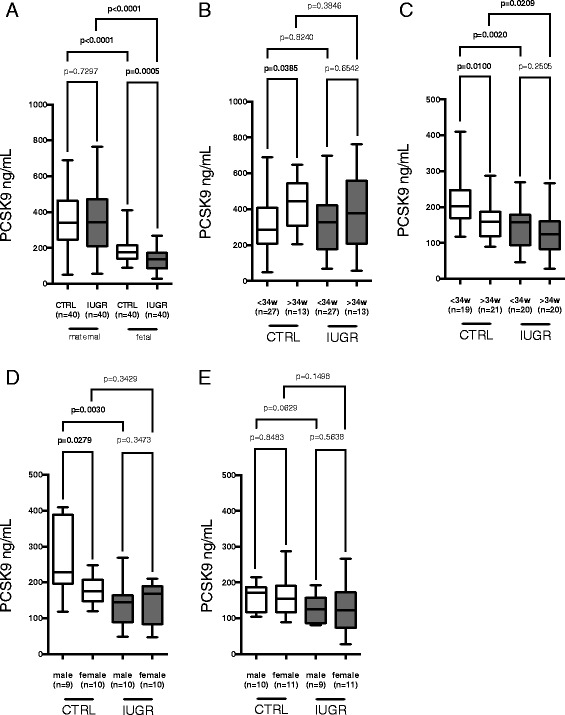
**a**-**e** Maternal and fetal serum PCSK9 concentrations of IUGR and CTRL in gestational age and gender subgroups presented as Tukey’s box plots. **a** Maternal and fetal PCSK9 concentration comparing IUGR and CTRL. **b** Maternal PCSK9 concentrations in IUGR and CTRL sampled between 24 and 34 versus after 34 weeks of gestation. **c** Fetal PSCK9 concentrations in IUGR and CTRL of those born between 24 and 34 versus after 34 weeks of gestation. **d**-**e** Fetal PCSK9 concentrations grouped by gestational age at delivery (**d**) before and **e**) after 34 weeks of gestation) and fetal gender

**Fig. 2:**
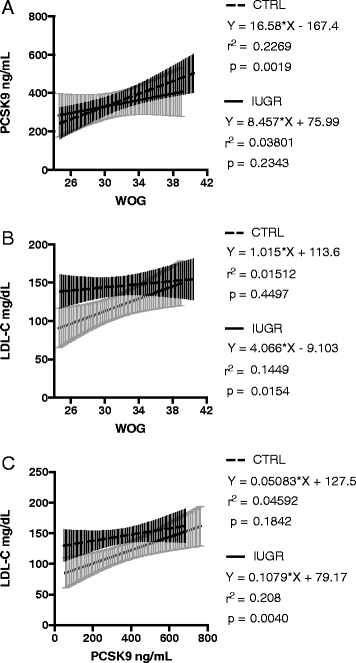
**a**-**c** Linear regression analyses to estimate the association between maternal PCSK9 and LDL-C serum concentrations, and gestational age at blood sampling. Displayed are regression lines and 95% Confidence Intervals separately for the IUGR and the CTRL group. **a** PCSK9 and gestational age, **b**) LDL-C and gestational age, **c**) PCSK9 and LDL-C. WOG = week of gestation

PCSK9 concentrations in maternal blood were twice as high as in the fetal blood (Fig. 1a). Within the CTRL group PCSK9 concentration levels were positively associated with gestational age at sampling (Fig. 2a). However, LDL-C levels in the CTRL group remained remarkably constant from 24 weeks of gestation onward until term, while increasing LDL-C levels could be observed in the IUGR group (Fig. 2b).

There were no significant differences in maternal PCSK9 serum concentrations between IUGR and CTRL (Fig. 1a). Multivariate regression analyses revealed that both, maternal PCSK9 levels and the study group ‘IUGR’ independently predicted maternal LDL-C levels (adjusted *R* ^2^ = 0.2526; PCSK9 coefficient *b*_*i*_ = 0.0938, standard error s_*bi*_ = 0.0217, r_partial_ = 0.4420, *t*-value = 4.323, *p* < 0.0001, IUGR coefficient *b*_*i*_ = −28.7631, standard error s_*bi*_ =8.8658, r_partial_ = −0.3468, *t*-value = −3.244, *p* < 0.0017). Adjustment for other clinical characteristics like maternal age, BMI, and smoking status, or gestational age did not improve the predictive value. However, separate subgroup analyses confirmed an association of PCSK9 and LDL-C in the IUGR group but not in the CTRL group (Fig. 2c). In a logistic regression analysis maternal LDL-C levels but not PCSK9 remained an independent predictor for IUGR (Odds Ratio = 0.985 (95% CI 0.974–0.996), *p* = 0.0086).

### PCSK9 in the fetal compartment

**Fig. 3:**
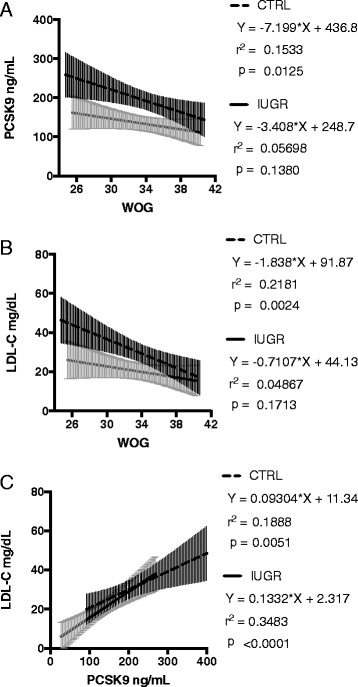
**a**-**c** Linear regression analyses to estimate the association between fetal PCSK9 and LDL-C serum concentrations, and gestational age at delivery. Displayed are regression lines and 95% Confidence Intervals separately for the IUGR and the CTRL group. **a** PCSK9 and gestational age, **b** LDL-C and gestational age, **c** PCSK9 and LDL-C. WOG = week of gestation

In contrast to findings in the maternal compartment, fetal PCSK9 and LDL-C concentration levels in the CTRL group were negatively associated with gestational age at delivery (Fig. 3a and b). Mean fetal PCSK9 concentration in IUGR was 35% lower than in CTRL (Fig. 1a). The effect persisted when subgrouping into early and late onset IUGR according to gestational age at birth, e. g. neonates born between 24 and 34 weeks versus those born after 34 weeks of gestation (Fig. 1c). Further subgrouping according to fetal gender revealed highest PCSK9 concentrations in male neonates of the CTRL group born before 34 weeks of gestation (Fig. 1d). Significant differences between IUGR and CTRL remained consistent only for this subgroup (Fig. 1d and e).

In multivariate regression analyses fetal PCSK9 levels remained an independent predictor of fetal LDL-C concentrations (adjusted *R* ^*2*^ = 0.2929; *b*_*i*_ = 0.1156, s_*bi*_ =0.020, r_partial_ = 0.5494, *t*-value = 5.81, *p* < 0.0001). Adjustment for other clinical characteristics like gestational age or fetal gender did not increase the predictive value. Subgroup analyses confirmed a positive association of PCSK9 and LDL-C in both groups (Fig. 3c). In logistic regression analysis fetal PCSK9 was predictive for IUGR (Odds Ratio = 0.986 (95% CI 0.977–0.994), *p* = 0.0015). Considering fetal LDL-C levels in the model did not improve the predictive value.

### Placental PCSK9 and LDLR expression patterns

**Fig. 4:**
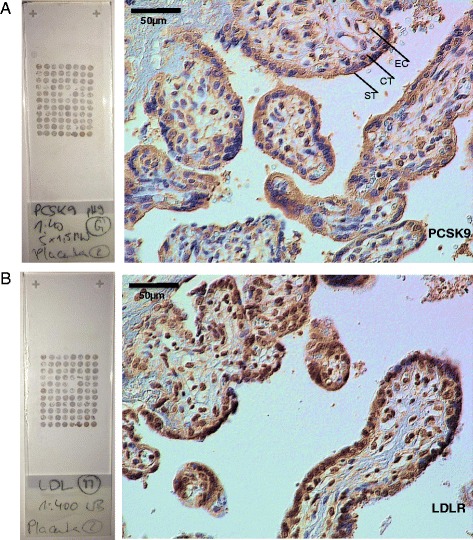
**a**-**b** Sample of a tissue microarray slide and representative high power field (×400) of A) PCSK9 and B) LDLR immunohistochemistry. Staining patterns were similar in CTRL and IUGR. However, staining intensity was higher for the LDLR in IUGR (as shown in Fig. 5). CT = cytotrophoblast, ST = syncytiotrophoblast, EC = endothelial cell

**Fig. 5:**
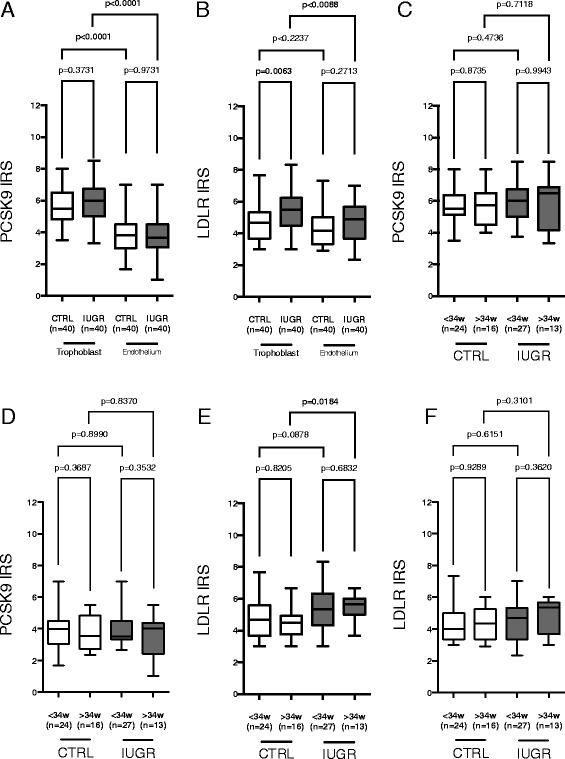
**a**-**f** Placental (**a**) PCSK9 and (**b**) LDLR immunoreactivity score (IRS) for IUGR and CTRL presented as Tukey’s box plots separately for the maternoplacental (trophoblast) and fetoplacental (endothelium) interface. Analyses were further performed for subgroups according to gestational age at delivery (before and after 34 weeks of gestation), and separately for trophoblast (**c**) PCSK9, **e**) LDLR) and endothelium (**d**) PCSK9, **f**) LDLR)

Staining for PCSK9 was predominantly found in trophoblast (cytotrophoblast and syncytiotrophoblast) and endothelial cells, and to a minor degree in villous stroma cells (Fig. 4a). No differences have been found in PCSK9 expression patterns between IUGR and CTRL when estimating IRS separatley for trophoblast and the endothelium (Fig. 5a).

LDLR expression could be observed throughout all compartments of the placenta, the trophoblast, placental stroma cells, and the endothelium (Fig. 4b). LDLR expression was higher in the trophoblast layer of IUGR as compared to CTRL placentas (Fig. 5b). When subgrouping according to gestational age at delivery the higher LDLR expression in IUGR consistently could be found in placentas delivered after 34 weeks of gestation (Fig. 5e).

In correlation analyses LDLR expression in trophoblasts over all was tendentielly negatively associated with maternal LDL-C (*r* = −0.22, *p* = 0.05), while LDLR expression in endothelial cells was positively associated with fetal LDL-C levels (*r* = 0.24, *p* = 0.036). No other major associations have been observed between placental PCSK9 and LDLR expression patterns and clinical or biochemical parameters, respectively.

## Discussion

To the best of our knowledge this is the first analysis of PSCK9 concentration and expression patterns in the maternal, fetal, and placental compartment in a well-defined IUGR cohort.

We confirmed lower LDL-C concentrations in maternal and fetal blood in IUGR as compared to CTRL. PCSK9 independently predicted LDL-C concentration levels in both compartments. However, while maternal PCSK9 levels did not differ significantly between IUGR and CTRL, we found significant lower concentration levels of PCSK9 in umbilical cord blood of IUGR as compared to the CTRL group, suggesting that independent regulatory metabolic pathways are activated in the mother and in the fetus. In addition, the placenta as the linking organ showed higher LDLR expression patterns in IUGR when compared to CTRL while we found no significant difference in PCSK9 expression.

Few studies have been performed to analyze PCSK9 expressions in pregnancy, so far. Interestingly, Peticca et al. recently showed that PCSK9 did not correlate with LDL-C levels in uncomplicated third-trimester pregnancies. The physiological disruption of this association during pregnancy suggests that, unlike in women in the non-pregnant state, other effects are superior to PCSK9 in controlling maternal LDL-C levels [17]. The present study partially confirmed their findings. Maternal PCSK9 levels were associated with LDL-C levels only in the IUGR group but not in uncomplicated pregnancies. This suggests an inadequate adaptation of maternal metabolism in pregnancy when complicated by IUGR. The placenta plays a crucial role in the pathogenesis of IUGR and, as a main source of steroid hormones during pregnancy, is highly dependent on cholesterol supply [2]. Recently, Dubé et al. showed that mRNA and protein of PCSK9 and its target protein LDLR is expressed in the human placenta suggesting an impact of PCSK9 on the regulation of placental LDL uptake [18]. We questioned whether differences in the activation of metabolic pathways could be confirmed in the placenta of IUGR and therefore analyzed expression patterns by immunohistochemistry using tissue microarrays. This method allows standardizing staining techniques and estimating expression patterns by directly comparing 80 individual samples within a single slide. The advantage of immunohistochemistry over other techniques for protein expression determination is the possibility to estimate cell-staining intensities in different compartments of the placenta, e. g. the maternoplacental (trophoblast), and the fetoplacental surface (endothelium) [21]. We could neither detect any differences in expression patterns of PCSK9 in the trophoblast nor in the endothelium of IUGR placentas when compared to CTRL. However, we found a stronger expression of LDLR in trophoblasts of IUGR than in CTRL placentas at least after 34 weeks of gestation. This is in agreement with other publications. Stepan et al. found LDLR mRNA expression to be upregulated in IUGR placentas [26]. Wadsack et al. reported a higher placental LDLR protein expression [9]. The authors suggested that compensatory mechanisms to the lower maternal LDL concentration might be activated in order to guarantee fetal cholesterol supply. Dubé et al. recently reported on lower maternal LDL-C levels in pregnancies complicated by gestational diabetes as compared to normal pregnancy. Yet, in their study they found lower placental PCSK9 expression levels in the diabetes group [18]. The contradiction to our results may be explained by different methods (Dubé et al. used western blot to quantify PCSK9 expression. This method does not take into account differences in placental morphology and composition between study groups) or by different pathways being involved in the regulation of LDL-C metabolism in the two entities. While PCSK9 acts posttranscriptionally by degradation of the LDLR, higher mRNA levels in IUGR placentas as observed by Stepan et al. suggest a transcriptional regulation [26].

In the fetal compartment we found PCSK9 concentration levels positively associated with LDL-C levels which is in agreement with the literature [16, 17]. In IUGR circulating PCSK9 concentrations were lower when compared to CTRL. Two other studies analyzed fetal cord blood PCSK9 concentrations in small for gestational age neonates (SGA). Neither of the studies found any differences in PCSK9 concentration levels between their SGA and control groups [16, 17]. The terms ‘SGA’ and ‘IUGR’ are often used synonymeously which may lead to misinterpretation and may explain the mismatch to our results. Peticca et al. and Araki et al. defined SGA by birth weight centile only. We recently pointed out that it is mandatory to differentiate between constitutional SGA and IUGR by specific antenatal sonographic criteria in addition to an estimated low fetal weight [7, 19]. Moreover, it has been suggested to consider two different phenotypes when studying IUGR, that can be devided according to the gestational age when IUGR is diagnosed: early onset (before 34 weeks) and late onset (after 34 weeks of gestation) IUGR [23, 24]. Equally important, evidence suggests a sex-specific fetal response to maternal diseases during pregnancy [27]. In the present study, both gestational age and fetal gender significantly influenced fetal PCSK9 levels. When subgrouping patients accordingly differences between IUGR and CTRL remained significant only for male fetuses delivered before 34 weeks of gestation (early onset IUGR). Yet, the number of patients in each subgroup is too small to draw a final conclusion and further studies are necessary. However, our results are in line with the fact that in response to adverse in-utero insults, male versus female infants have greater disadvantages in pregnancy outcome. One explanation may be that the placenta of the female fetus protects them from adverse prenatal events [28]. Possibly, however, the growing male fetus responds differently to sex hormones like estrogens or progesterons during the in-utero periode. We recently demonstrated that in addition to reduced LDL-C concentrations estrogen and progesterone levels are lower in IUGR pregnancies [2]. Indeed, estrogen influences PCSK9 levels and hence cholesterol catabolism [29]. Whether this mechanism selectively affects fetal growth in the male fetus remains to be clarified.

Our study has some limitations. We cannot exclude that cord blood and placental parameters within the CTRL group are biased by factors that may lead to spontaneous onset of premature labor. However, gestational age is one of the main factors affecting cord blood composition [7]. Hence, the study groups need to be controlled for this issue. Due to obvious ethical and practical reasons fetal blood and placental samples cannot be obtained in healthy uncomplicated and ongoing pregnancies antenatally. Moreover, differences in the treatment regimes between IUGR and CTRL mothers may have an influence on maternal and/or fetal metabolism. For example, mothers with spontaneous onset of preterm labor or preterm premature rupture of membranes receive antibiotics and tocolytic agents, while IUGR mothers do not. Stress also influences lipid parameters, and fetal distress is a common medical indication for delivery in patients with IUGR.

## Conclusion

In conclusion, the results of the present study suggest that during pregnancy LDL-C metabolism is under control of PCSK9 at least in the fetal compartment. In the maternal compartment PCSK9 was rather mildly associated, and other mechanisms that control LDL-C levels remain to be identified. Lower fetal PCSK9 concentration levels in IUGR as compared to CTRL might be the result of altered metabolic adaptation of the fetus in response to a pathological process during preganancy, and hence might play a crucal role in the pathophysiology of IUGR. Since we found fetal PCSK9 concentrations being considerably dependent on gestational age and gender, particular individuals of IUGR might be identified in the future who benefit from pharmaco-therapy targeting the fetal PCSK9 pathway. Yet this has to be elucidated in further studies.

## Abbrevations

ARED: Absent or reversed end-diastolic blood flow
CTRL: Control group
HDL-C: High density lipoprotein-cholesterol
IRS: Immunoreactivity score
IUGR: Intrauterine growth restriction
LDL-C: Low density lipoprotein-cholesterol
LDLR: LDL-receptor
PCSK9: Proprotein Convertase Subtilisin/Kexin-Type 9
SGA: Small for gestational age
TC: Total cholesterol
TG: Triglyceride
